# Brain tumor diagnostic model and dietary effect based on extracellular vesicle microbiome data in serum

**DOI:** 10.1038/s12276-020-00501-x

**Published:** 2020-09-16

**Authors:** Jinho Yang, Hyo Eun Moon, Hyung Woo Park, Andrea McDowell, Tae-Seop Shin, Young-Koo Jee, Sungmin Kym, Sun Ha Paek, Yoon-Keun Kim

**Affiliations:** 1 MD Healthcare R&D Institute, Seoul, Republic of Korea; 2 Department of Health and Safety Convergence Science Introduction, Korea University, Seoul, Republic of Korea; 3 Department of Neurosurgery, Clinical Research Institute, Seoul National University Hospital, Seoul, Republic of Korea; 4 Department of Internal Medicine, Dankook University College of Medicine, Cheonan, Korea; 5 Department of Internal Medicine, Inje University Haeundae Paik Hospital, Inje University College of Medicine, Busan, Republic of Korea; 6 Department of Neurosurgery, Cancer Research Institute, Hypoxia Ischemia Disease Institute, Seoul National University, Seoul, Republic of Korea

**Keywords:** Diagnostic markers, Machine learning

## Abstract

The human microbiome has been recently associated with human health and disease. Brain tumors (BTs) are a particularly difficult condition to directly link to the microbiome, as microorganisms cannot generally cross the blood–brain barrier (BBB). However, some nanosized extracellular vesicles (EVs) released from microorganisms can cross the BBB and enter the brain. Therefore, we conducted metagenomic analysis of microbial EVs in both serum (152 BT patients and 198 healthy controls (HC)) and brain tissue (5 BT patients and 5 HC) samples based on the V3–V4 regions of 16S rDNA. We then developed diagnostic models through logistic regression and machine learning algorithms using serum EV metagenomic data to assess the ability of various dietary supplements to reduce BT risk in vivo. Models incorporating the stepwise method and the linear discriminant analysis effect size (LEfSe) method yielded 12 and 29 significant genera as potential biomarkers, respectively. Models using the selected biomarkers yielded areas under the curves (AUCs) >0.93, and the model using machine learning resulted in an AUC of 0.99. In addition, *Dialister* and *[Eubacterium] rectale* were significantly lower in both blood and tissue samples of BT patients than in those of HCs. In vivo tests showed that BT risk was decreased through the addition of sorghum, brown rice oil, and garlic but conversely increased by the addition of bellflower and pear. In conclusion, serum EV metagenomics shows promise as a rich data source for highly accurate detection of BT risk, and several foods have potential for mitigating BT risk.

## Brain cancer: Investigating the gut microbiome

The gut microbiome affects brain health via tiny packets of microbial metabolites called extracellular vesicles (EVs) that are small enough to pass through the blood–brain barrier. The brain was thought to be sheltered from the microbiome’s effect on health by this barrier, which blocks microbes from entering the brain via the blood. Yoon-Keun Kim at MD Healthcare Inc. and Sun Ha Paek at Seoul National University, both in Seoul, South Korea, and co-workers compared the EVs in the blood of brain cancer patients and healthy individuals, and found the two groups harbored different types of microbes. Feeding mice brown rice oil, sorghum, and garlic markedly shifted their EV profiles towards those of the healthy microbiome. These results provide a foundation for investigating new methods for brain cancer risk assessment and dietary interventions to reduce that risk.

## Introduction

The human microbiome is the collection of genes contributed by the total microbial community in our body and has been associated with human health and disease. Despite the majority of the bacteria in our bodies residing in the gastrointestinal (GI) tract, the characteristics and activity of the microbiome have far reaching effects on metabolism, immune function, and carcinogenic activity^1–3^. Recent interest has been particularly paid to understanding the emerging relationship between our microbiome and cancer. Through integration of the holobiont paradigm in oncological research, recent studies have demonstrated a complex relationship between the microorganisms occupying our intestinal tract and carcinogenesis. The gut microbiota has been reported to impact the tumor macroenvironment through modulation of host immune and neuroendocrine factors^4^. The microbiome exerts tremendous influence on immune function, development and response, with over 70% of the immune cells in our body residing in our gut^5,6^. Furthermore, certain microorganisms have been identified to have cancer-promoting effects, while others exert inhibitory effects on cancer growth by boosting the body’s immune response and diminishing immune evasion of cancer cells^7^. Therefore, the complex dynamics between the gut microbiome and cancer micro- and macroenvironments must be elucidated to better understand the multifaceted manner in which cancer develops and progresses.

While the field of microbiome research has made tremendous strides in the 21st century, the nanosized extracellular vesicles (EVs) released by bacteria and archaea remain a relatively underexplored area of study. Microbial EVs are typically 20–200 nm in diameter and composed of a lipid bilayer encapsulating various proteins, nucleic acids and metabolites derived from the parent microbial cell^8^. The biogenesis of EVs is closely regulated in both Gram-negative and Gram-positive commensal bacteria, and EVs are generated throughout cell proliferation as ectosomes and cell death as apoptotic bodies^9^. Microbial EVs have been shown to travel distally throughout the body and are detectable in a variety of samples, including stool, urine, serum, and tissue, and elicit a variety of immunological effects in vitro and in vivo^9,10^. Previously, we reported the effective use of microbial EVs for cancer diagnostics using human serum^11^. Furthermore, *Lactobacillus rhamnosus* EVs have been shown to exert a cytotoxic effect on hepatic cancer cells^12^. A previous meta-analysis of 22 cohort and case–control studies revealed that obesity is a risk factor for meningiomas, gliomas, and brain and central nervous system (CNS) tumors in females and meningiomas in males^13^. Our group recently reported that dietary intervention with several different grains was capable of ameliorating high-fat diet-induced microbiome-associated colorectal cancer risk in vivo^14^. Therefore, we sought to apply this methodology to determine the role of the EV microbiome in brain cancer by analyzing microbial EVs circulating in the serum and brain tissue of brain tumor (BT) patients and healthy control (HC) subjects.

As nanosized microbial vesicles are able to enter circulation and interact with a variety of host sites via distal intercellular communication, microbial EVs play an immunomodulatory role in the cancer macroenvironment in addition to the cancer microenvironment. BTs are a particularly difficult area to directly link to the microbiome, as microorganisms themselves are generally incapable of crossing the blood–brain barrier (BBB). The BBB also presents a major hurdle for clinical applications of chemotherapy medications such as doxorubicin and paclitaxel for glioma treatment due to the p-glycoprotein drug efflux mechanisms associated with the BBB^15^. However, previous studies recently revealed that nanosized EVs released from commensal microorganisms are capable of crossing the BBB and entering the brain^16,17^.

Therefore, as the microbiome is known to affect the body’s susceptibility to cancer and has a direct link to the brain via the gut–brain axis, we sought to elucidate the relationship between the EV microbiome and BTs. We conducted metagenomic analysis of microbial EVs isolated from both serum and brain tissue collected from BT patients and HC subjects to determine significant microbiome alterations in the cancer macroenvironment and microenvironment. We then developed intelligent diagnostic models at the genus level using serum EV metagenomic data and machine learning algorithms. The resulting diagnostic models were used to assess the ability of a variety of grain, protein, lipid and vegetable dietary supplements to reduce BT risk induced by consumption of a high-fat diet (HFD). Our findings support the role of microbial EVs and diet in the tumor micro- and macroenvironments and the need for further assessment for future diagnostic and therapeutic development.

## Materials and methods

### Subjects and sample collection

In total, serum samples of 152 BT patients and 198 HC subjects were obtained from Seoul National University Hospital and Inje University Haeundae Hospital, respectively. In addition, tissue samples were obtained from 5 BT patients and 5 HC subjects enrolled from Seoul National University Hospital. Each BT clinical subject presented symptoms leading them to visit the hospital for treatment. Healthy control subjects were screened through a general health examination. The present study was approved by the Institutional Review Board of Seoul National University Hospital (IRB No. H-1009-025-331) and Inje University Haeundae Hospital (IRB No. 1297992-2015-064). All methods in this study were conducted in accordance with the approved guidelines, and informed consent was obtained from all clinical subjects.

All collected human serum samples were transferred to serum separator tubes (SSTs) and then centrifuged at 3000 rpm for 15 min at 4 °C. All brain tissue samples were frozen in liquid nitrogen and stored at −80 °C for analysis.

### In vivo mouse study model

All mice used in this study were female C57BL/6 mice at 6 weeks of age (Orient Bio Inc., Seongnam, Korea). Mice were housed and maintained under standard laboratory conditions of 22 ± 2 °C and 50 ± 5% humidity under 12-h day and night cycles throughout the course of the in vivo study. The animal study was approved by the Institutional Animal Care and Use Committee of Chung-Ang University (Approval No. 2018-00057). All methods in this animal study were conducted in accordance with the approved guidelines.

### Evaluation of dietary effects

To analyze dietary effects, in vivo sampling of mouse serum was conducted before and after a dietary intervention. First, mice were randomly divided into two groups (*n* = 60): a group fed a regular chow diet (RCD) and a group fed a HFD. Second, mice were randomly divided into 30 groups (*n* = 5) including an RCD group, HFD group, and HFD + group fed an HFD supplemented with adlay, glutinous rice, nonglutinous rice sorghum, buckwheat, brown rice, acorn, mung bean, roasted bean, fermented bean, mealworm, perilla (*Perilla frutescens var. japonica Hara*), perilla oil, brown rice oil, sesame oil, broccoli, garlic, ginger, turmeric (*Curcuma longa Linne*), lotus root, cabbage, bellflower (*Platycodon grandiflorum*), onion, pumpkin, pear, grape, kelp, or shiitake mushroom. Mice within the RCD control group were fed regular chow containing 18% dietary fat (Research Diets, Inc., New Brunswick, NJ, USA) for 4 weeks. Mice in the HFD group were fed a 60% fat diet (Research Diets, Inc), and diet powder (100 µg) or oil (100 µL) was orally administered once every day for 4 weeks. At the conclusion of the 4-week study period, all mice were sacrificed, and serum was collected for EV microbiome compositional assessment.

### EV DNA extraction and sequencing

To extract EVs from serum and tissue samples, centrifugation, filtering, and boiling methods were performed as described in our previous study^11^. Serum and tissue EV DNA were extracted using a DNeasy PowerSoil kit (QIAGEN, Germany). Finally, the extracted EV DNA in each sample was quantified using QIAxpert (QIAGEN). Isolated EV microbial genomic DNA was amplified by targeting the 16S V3–V4 hypervariable regions. The libraries were prepared using PCR products, and all amplicons were sequenced using a MiSeq instrument (Illumina, USA).

### Metagenomic analysis of microbial EV composition

Taxonomic assignment was performed by the profiling program MDx-Pro ver. 2 (MD Healthcare, Korea). Briefly, paired-end reads were filtered according to the barcode, and primer sequences were trimmed using Cutadapt (version 1.1.6) and then merged with CASPER. To obtain high-quality sequencing reads, sequences with read lengths under 350 bp or over 550 bp and with Phred quality scores below 20 were discarded. The VSEARCH de novo clustering method was used to assign operational taxonomic units (OTUs) to the genus level with a 97% similarity threshold. OTUs containing 1 sequence in only one sample were excluded from further analysis. Subsequently, taxonomic assignment was conducted to the species level using UCLUST and QIIME 1.9.1 against the Silva 132 database under default parameters. If clusters could not be assigned at the genus level due to insufficient taxonomic information in the database, the taxon was assigned to the next highest level indicated in parentheses. Brackets around the taxon name represent an unverified, suggested taxonomic assignment based primarily on whole genome phylogeny within the genomic database.

### Predictive diagnostic model development

For the development of a BT predictive diagnostic model, we considered the relative abundances of OTUs at the genus level as model variables. First, we selected candidate biomarkers with *p*-values below 0.01, fold-changes >2, and average relative abundances >0.1%. Biomarkers included as model variables were selected by one of four methods that were then compared to determine the model with the highest area under the curve (AUC), sensitivity, specificity, and accuracy. The first method (M1) used stepwise selection for which the Akaike information criterion (AIC) was used for comparison among predictive diagnostic models with differing variables. The second method (M2) incorporated age and sex as covariates in addition to stepwise selection methodology. The third selection method (M3) used linear discriminant analysis (LDA) and LDA effect size (LEfSe) algorithms for biomarker discovery, while the fourth method (M4) included age and sex as covariates in addition to incorporating biomarkers selected using LEfSe. In addition, the fifth model (M5) was calculated by a machine learning algorithm based on the gradient boosting machine (GBM) ensemble method. The GBM was incorporated in the modeling process using the Gradient Boosting Regressor of scikit-learn (version 0.21.3) in python (version 3.6.9). After variable selection, the predictive diagnostic model was calculated using logistic regression with training and test sets established at an 80:20 ratio for model validation.

### Statistical analysis

Significant differences in age between the BT and control groups were determined through Student’s *t*-test and Wilcoxon rank-sum test, respectively. A chi-square test was performed to determine any significant difference between the groups based on sex. To analyze alpha diversity, Chao1, Shannon index, and Simpson index were assessed. Chao1 corrects for observed richness, and the Shannon index considers species evenness and relative abundance in each sample. The Simpson index evaluates both evenness and richness by considering the total number of species and their abundance in a community^18^. Principal coordinate analysis (PCoA) was conducted to determine individual taxa-level clustering of groups based on Bray–Curtis dissimilarity distance. To analyze the difference in microbiome composition between the HC and BT groups, Student’s *t*-test was performed. LEfSe was also used to determine significant, differentially abundant genera between the clinical groups for the selection of biomarkers with statistical and biological significance. The LEfSe algorithm utilized the Wilcoxon rank-sum test and linear discriminant analysis (LDA) with the cut-off LDA score (log10) set as 2. The results were considered significant when *p*-values were <0.05 (*p* < 0.05), and all analyses were conducted using R version 3.6.1.

## Results

### Clinical characteristics of subjects

Through assessment of the clinical characteristics of the HC and BT subject groups, it was determined that there was a significant difference in age between the two groups (*p* < 0.001). The HC subjects ranged from 40 to 78 years of age with a mean age of 59.7 (SD 10.5) years, while the BT subjects yielded a mean age of 51.5 (SD 14.2) ranging from 16 to 81 years (Table 1).

**Table 1:** Subjects’ clinical characteristics.

	Serum			Tissue		
	HC (*n* = 198)	BT (*n* = 152)	*p*-Value	HC (*n* = 5)	BT (*n* = 5)	*p*-Value
Sex (M/F)	119/79	87/65	0.893	3/2	3/2	1.000
Age (mean ± SD)	59.7 ± 10.5	51.5 ± 14.2	<0.001	46.8 ± 11.9	54.0 ± 14.6	0.548
Pathology glioma (WHO grade III/IV)		107 (15/92)			2 (–/2)	
Metastatic brain tumor		45			3	
EGFR (+)		75			–	
EGFR VIII (+)		26			–	
IDH1/2 mutation (+)		15			–	
MGMT methylation (+)		61			1	
Chromosome 1p/Chromosome 19q (+)		3/8			−/1	

*HC* healthy control subjects, *BT* brain tumor patients, *EGFR* epidermal growth factor receptor, *IDH* isocitrate dehydrogenase, *MGMT O*^6^-methylguanine DNA methyltransferase.

### Comparison of alpha and beta diversity between healthy controls and brain tumor patients

**Fig. 1:**
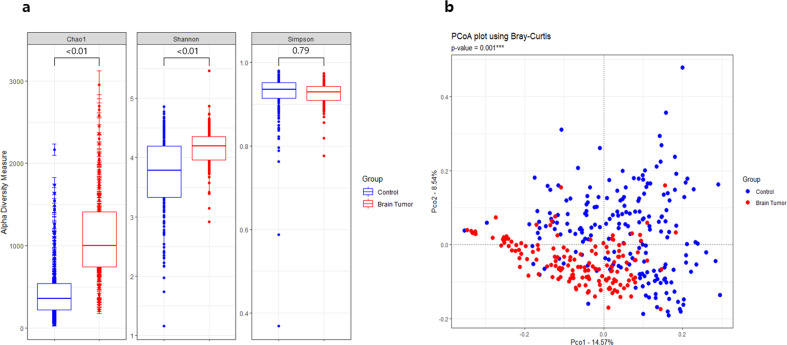
Alpha and beta diversity of serum in healthy control (HC) subjects and brain tumor (BT) patients. Differences in (**a**) alpha diversity (Chao1, Shannon, and Simpson index) and (**b**) beta diversity of the serum EV microbiome between the HC and BT groups using PCoA based on Bray–Curtis dissimilarity at the genus level.

The Chao1 index of species richness and Shannon index of bacterial community diversity were significantly higher in the BT group, whereas the difference in the Simpson index of diversity between the patient and control groups was not significant (Fig. 1a). PCoA was conducted, and all samples were plotted along the two principal coordinates (PCos) that accounted for the greatest dissimilarity between samples to evaluate the similarity between the HC and BT groups. At all taxa levels, significant clustering was observed between the two groups (*p* < 0.001) (Fig. 1b, Supplementary Fig. 1).

### Differences in microbial EV abundance in serum between healthy controls and brain tumor patients

**Fig. 2:**
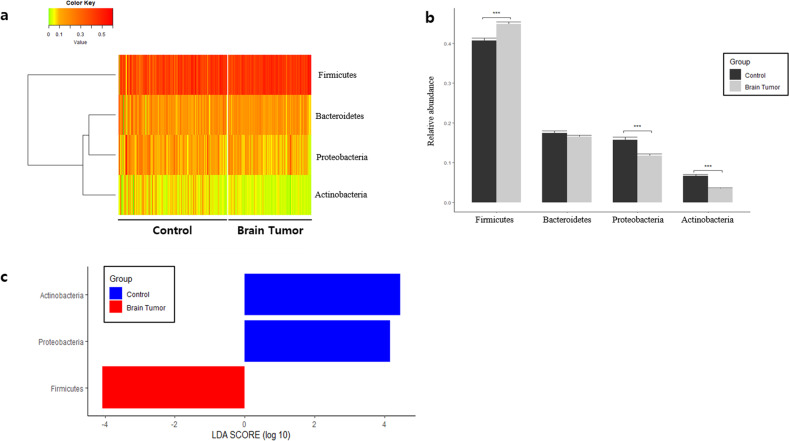
Differences in microbiome abundance between healthy control (HC) subjects and brain tumor (BT) patients in serum at the phylum level. **a** Heatmap of serum microbial EV abundance. **b** Major bacterial EVs and significant difference between the HC and BT groups by *t*-test (>1% in any group). **c** Significantly different bacterial EVs determined through LEfSe analysis (>4 log(LDA score)).

**Fig. 3:**
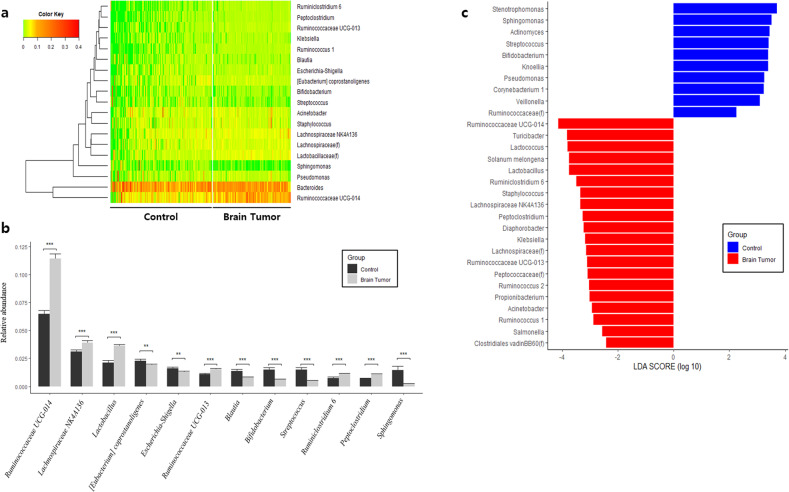
Differences in microbiome abundance between healthy control (HC) subjects and brain tumor (BT) patients in serum at the genus level. **a** Heatmap of serum microbial EV abundance. **b** Major bacterial EVs and significant difference between the HC and BT groups by *t*-test (>1% in any group). **c** Significantly different bacterial EVs determined through LEfSe analysis (>2 log(LDA score)).

At the phylum level, Firmicutes abundance was significantly lower in the control group than in the patient group, whereas Actinobacteria and Proteobacteria were higher (Fig. 2a, b). LEfSe analysis of phylum-level biomarkers yielded Actinobacteria, Proteobacteria, and Firmicutes as the only phyla with log(LDA score) values >4 (Fig. 2c). At the class level, Clostridia, Bacilli, Erysipelotrichia, Gammaproteobacteria, Actinobacteria, Alphaproteobacteria, and Negativicutes were significantly altered. Eight class-level biomarkers were determined with Actinobacteria yielding a log(LDA score) of 4.0, indicating statistically and biologically significant higher abundance in the control group (Supplementary Fig. 2a). At the order level, Clostridiales, Bifidobacteriales, Erysipelotrichales, Lactobacillales, Micrococcales, Sphingomonadales, and Selenomonadales were determined to be significantly altered between the healthy control and patient groups. LEfSe assessment at the order level revealed 13 taxa that were significantly different between the healthy control and patient groups, with Clostridiales being the most significantly altered with a log(LDA score) value of 3.9 (Supplementary Fig. 2b). At the family level, Ruminococcaceae, Lactobacillaceae, Peptostreptococcaceae, Erysipelotrichaceae, Lachnospiraceae, Streptococcaceae, Sphingomonadaceae, and Porphyromonadaceae were significantly altered. Family-level biomarker analysis using LEfSe revealed a total of 21 taxa, and Ruminococcaceae was the microbial EV family that differed the most between the patient and control groups with a log(LDA score) value >4.0 (Supplementary Fig. 2c). Finally, genus-level analysis revealed a multitude of significantly different taxa between the HC and BT groups (Fig. 3a). *Ruminococcaceae UCG-014, Lachnospiraceae NK4A136, Ruminococcaceae UCG-013, Lactobacillus, Ruminiclostridium 6*, and *Peptoclostridium* were significantly lower in the control group than in the BT group, whereas *[Eubacterium] coprostanoligenes, Escherichia-Shigella, Blautia, Bifidobacterium, Streptococcus*, and *Sphingomonas* were significantly higher (Fig. 3b). LEfSe analysis of genus-level serum EV microbiome composition yielded a total of 30 genera, with *Ruminococcaceae UCG-014* standing out with a log(LDA score) over 4.0 (Fig. 3c).

A total of 4, 9, 12, 18, and 29 taxa showed a proportion higher than 0.5% in either group and significant difference between the control and patient groups using a *t*-test at the phylum, class, order, family, and genus levels, respectively (*p* < 0.05).

### BT diagnostic model development based on serum microbial EV metagenomics

**Fig. 4:**
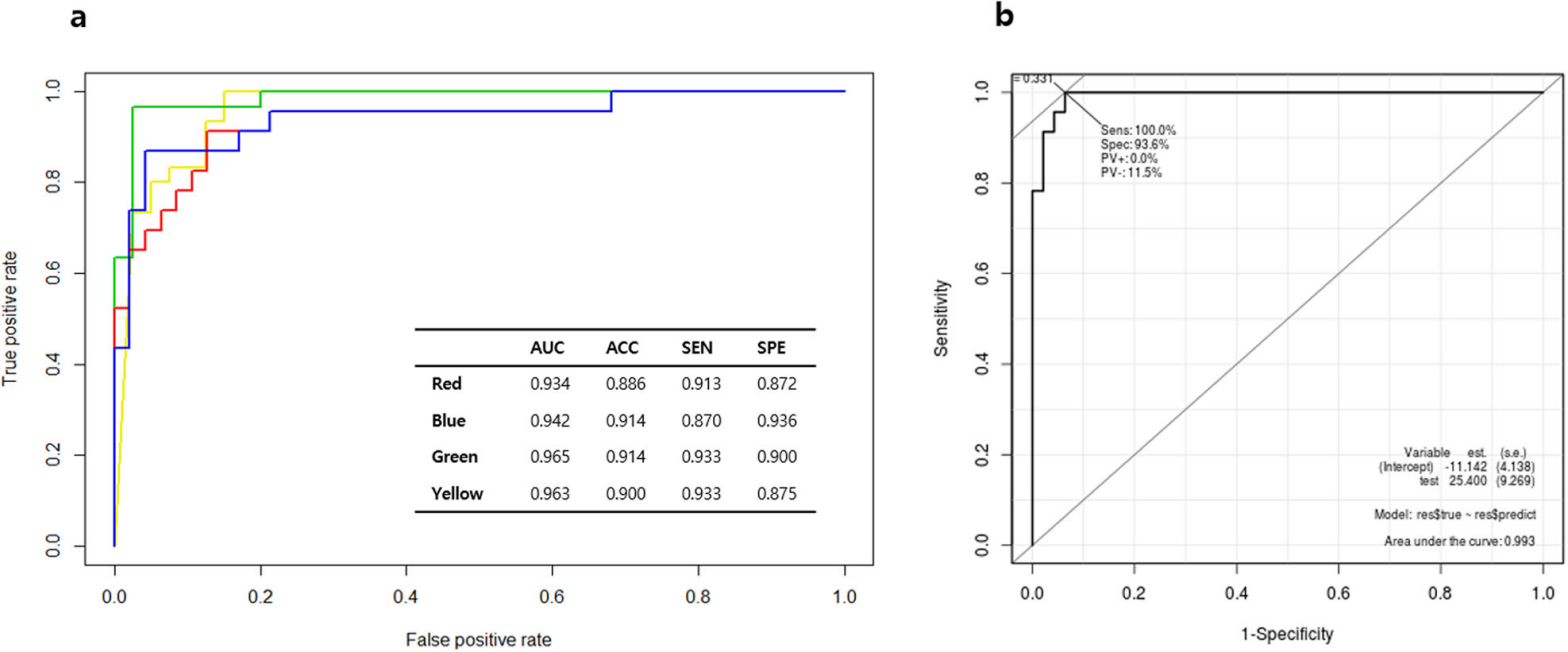
Brain tumor diagnostic models based on the serum EV microbiome at the genus level. Receiver operating characteristic (ROC) curves for (**a**) stepwise selection-based M1 (red) and M2 (blue) models, linear discriminant analysis effect size (LEfSe)-based M3 (green) and M4 (yellow) models, and (**b**) ROC curve for AI-based M5 model.

Using the serum microbial EV metagenomic profiles of the control and BT groups, diagnostic model sets were developed to determine BT risk in healthy subjects. The M1 and M2 models used logistic regression with stepwise selection and incorporated age and sex, respectively, as covariates in addition to stepwise selection. The M1 and M2 models were selected by the lowest AIC as optimal diagnostic models, while the M3 and M4 models applied logistic regression to selected biomarkers. The biomarkers of M3 were selected through LEfSe, while M4 included age and sex as covariates in addition to biomarkers selected using LEfSe. Finally, M5 was calculated by a machine learning algorithm based on the GBM method. Following stepwise selection and logistic regression analysis, the M1 and M2 models, which were optimized, yielded 12 significant microbial EV genera: *Stenotrophomonas, Knoellia, Sphingomonas, Solanum melongena, Parabacteroides, Actinomyces, Ruminiclostridium, Lactococcus, Turicibacter, Faecalibacterium, Streptococcus*, and *Bifidobacterium* (Fig. 3b). In addition, logistic regression utilizing biomarkers determined by LEfSe analysis for the M3 and M4 models revealed 29 different significant microbial EV genera: *Ruminococcaceae UCG-014, Lachnospiraceae NK4A136, Lactobacillus, Lachnospiraceae (f), Acinetobacter, Staphylococcus, Pseudomonas, Ruminococcaceae UCG-013, Klebsiella, Bifidobacterium, Ruminococcus 1, Streptococcus, Ruminiclostridium 6, Peptoclostridium, Sphingomonas, Clostridiales vadinBB60 (f), Turicibacter, Ruminococcaceae (f), Ruminococcus 2, Peptococcaceae (f), Diaphorobacter, Corynebacterium 1, Lactococcus, Propionibacterium, Solanum melongena, Actinomyces, Knoellia, Stenotrophomonas*, and *Veillonella* (Fig. 3c). In the case of M5, the relative abundance of the total microbial EV metagenomic information analyzed was input rather than specific biomarkers as features for analysis. Model performance using the test sets was evaluated based on the AUC, sensitivity, specificity, and accuracy of each method to determine the optimal BT diagnostic model. The resulting BT diagnostic models all yielded AUCs higher than 0.93, and the stepwise selection method showed a trend of lower AUC than LEfSe analysis for their given models (Fig. 4a). The model based on the GBM method showed the highest sensitivity, specificity, and AUC with values of 1.000, 0.936, and 0.993, respectively (Fig. 4b).

### Metagenomics of microbial EVs in brain tissue and comparison with serum microbiome

**Fig. 5:**
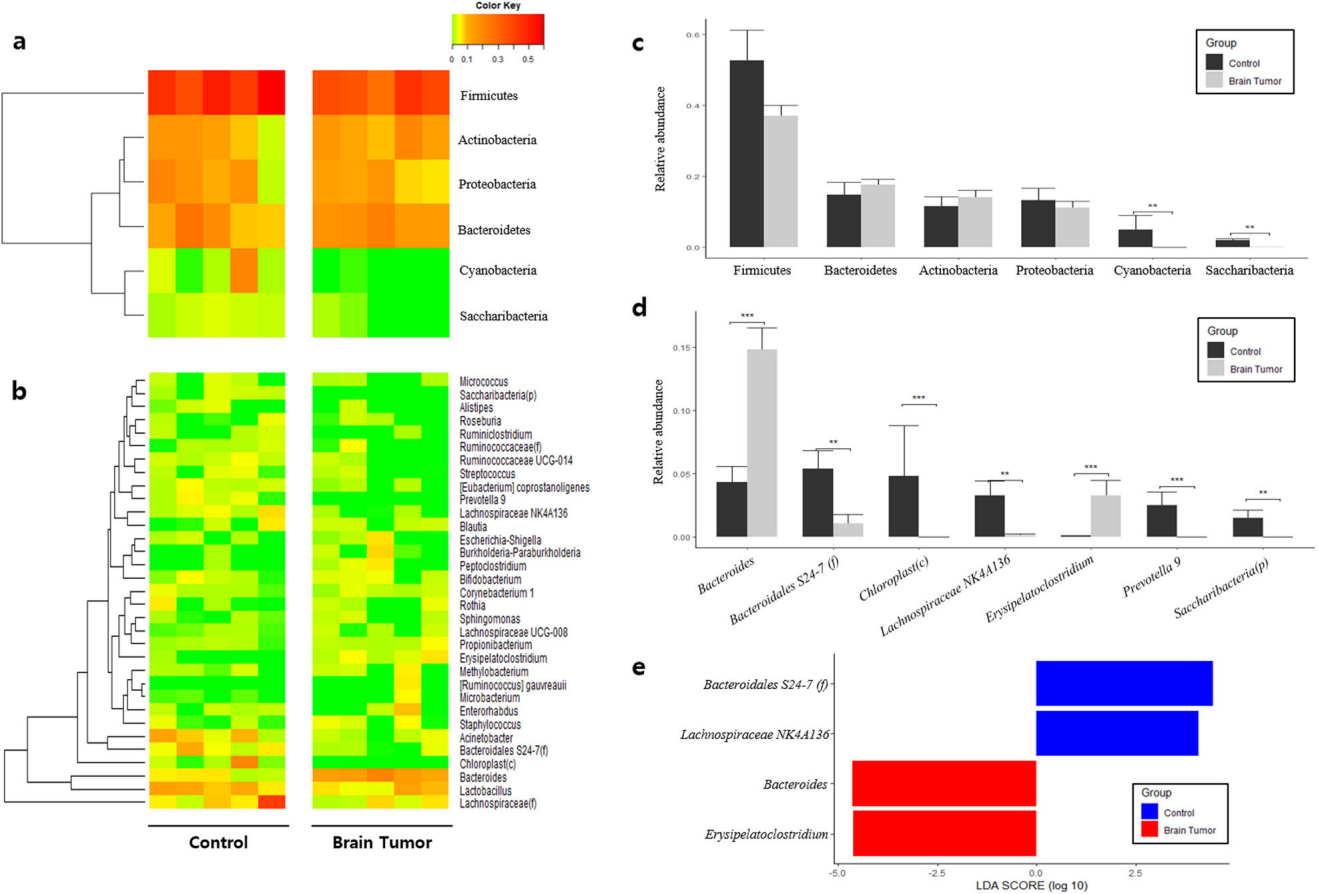
Differences in EV microbiome abundance between healthy control (HC) subjects and brain tumor (BT) patients in brain tissue at the phylum and genus levels. Heatmap of core microbial EV taxa in brain tissue at the **a** phylum and **b** genus levels. Major bacterial EVs of both clinical groups and significant differences between groups were assessed using a *t*-test at the **c** phylum and **d** genus levels. **e** Significantly different bacterial EV biomarkers selected via LEfSe at the genus level.

At the phylum level, Firmicutes, Bacteroidetes, Actinobacteria, and Proteobacteria were the most abundant phyla in all groups, accounting for over 80% of the tissue EV taxa in the patient and control groups. Cyanobacteria and Saccharibacteria were significantly higher in the control group than in the patient group (Fig. 5a, c). At the class level, Erysipelotrichia was significantly lower in the control group than in the patient group, whereas Clostridia, Saccharibacteria (p), and Chloroplast were significantly higher (Supplementary Fig. 3a). At the order level, Clostridiales, Chloroplast (c), and Saccharibacteria (p) were significantly enriched in the control group, whereas Erysipelotrichales was significantly depleted (Supplementary Fig. 3b). At the family level, Bacteroidaceae, Ruminococcaceae, Bacteroidales S24-7 group, Erysipelotrichaceae, Chloroplast (c), Prevotellaceae, and Saccharibacteria (p) were significantly altered between the clinical groups, and biomarkers discovered through LEfSe included Bacteroidales S24-7 group, Ruminococcaceae, Prevotellaceae, Bacteroidaceae, and Erysipelotrichaceae (Supplementary Fig. 3c). At the genus level, *Bacteroides* and *Erysipelatoclostridium* were significantly lower in the control group than in the BT group, whereas *Bacteroidales* S24-7 group, *Chloroplast (c), Lachnospiraceae NK4A136 group, Prevotella 9*, and *Candidatus Saccharimonas* were significantly higher (Fig. 5b, d). Genus-level LEfSe assessment revealed *Bacteroidales S24-7 group, Lachnospiraceae NK4A136, Bacteroides*, and *Erysipelatoclostridium* as significant BT biomarkers (Fig. 5e).

**Fig. 6:**
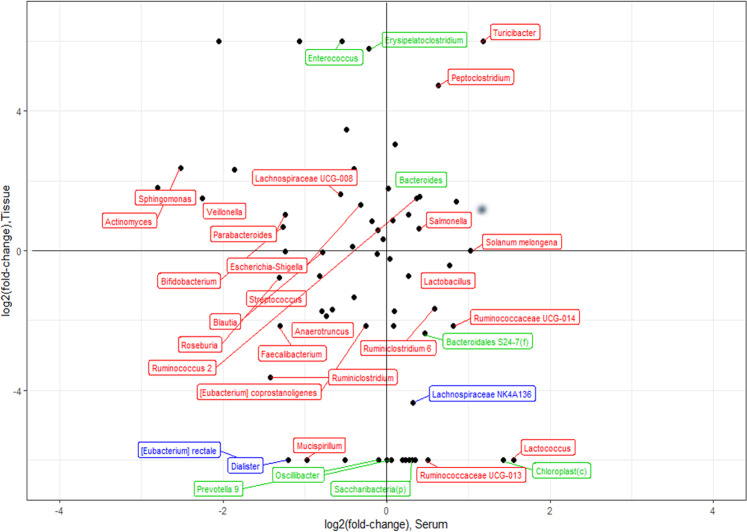
Fold-change between healthy control (HC) subjects and brain tumor (BT) patients in serum and tissue at the genus level. Red indicates genera of microbial EVs that were significantly different between the clinical groups in serum, while green represents those that were significant in tissue. Blue data points indicate microbial EV genera with significant differences in both serum and tissue samples.

To compare the microbiome compositional alterations of serum and tissue between controls and BT patients, fold-change of the microbial EV composition of serum and tissue obtained from the same individuals was analyzed. At the phylum level, Saccharibacteria in patients was significantly decreased in both serum and tissue (Supplementary Fig. 4a). At the class level, Erysipelotrichia was significantly increased in both serum and tissue samples collected from BT patients (Supplementary Fig. 4b). At the order level, Erysipelotrichiales was also significantly increased in both patient serum and tissue samples (Supplementary Fig. 4c). At the family level, Erysipelotrichiales was significantly higher and Prevotellaceae was significantly reduced in patient serum and tissue (Supplementary Fig. 4d). Finally, at the genus level*, [Eubacterium] rectale (E. rectale)* and *Dialister* were significantly decreased in both serum and tissue samples obtained from BT patients. In addition, *Lachnospiraceae NK4A136* was significantly lower in BT patient tissue, whereas it was significantly increased in patient serum compared to its levels in control tissue and serum samples (Fig. 6).

### Dietary effect on brain tumor risk based on the HFD mouse model

**Fig. 7:**
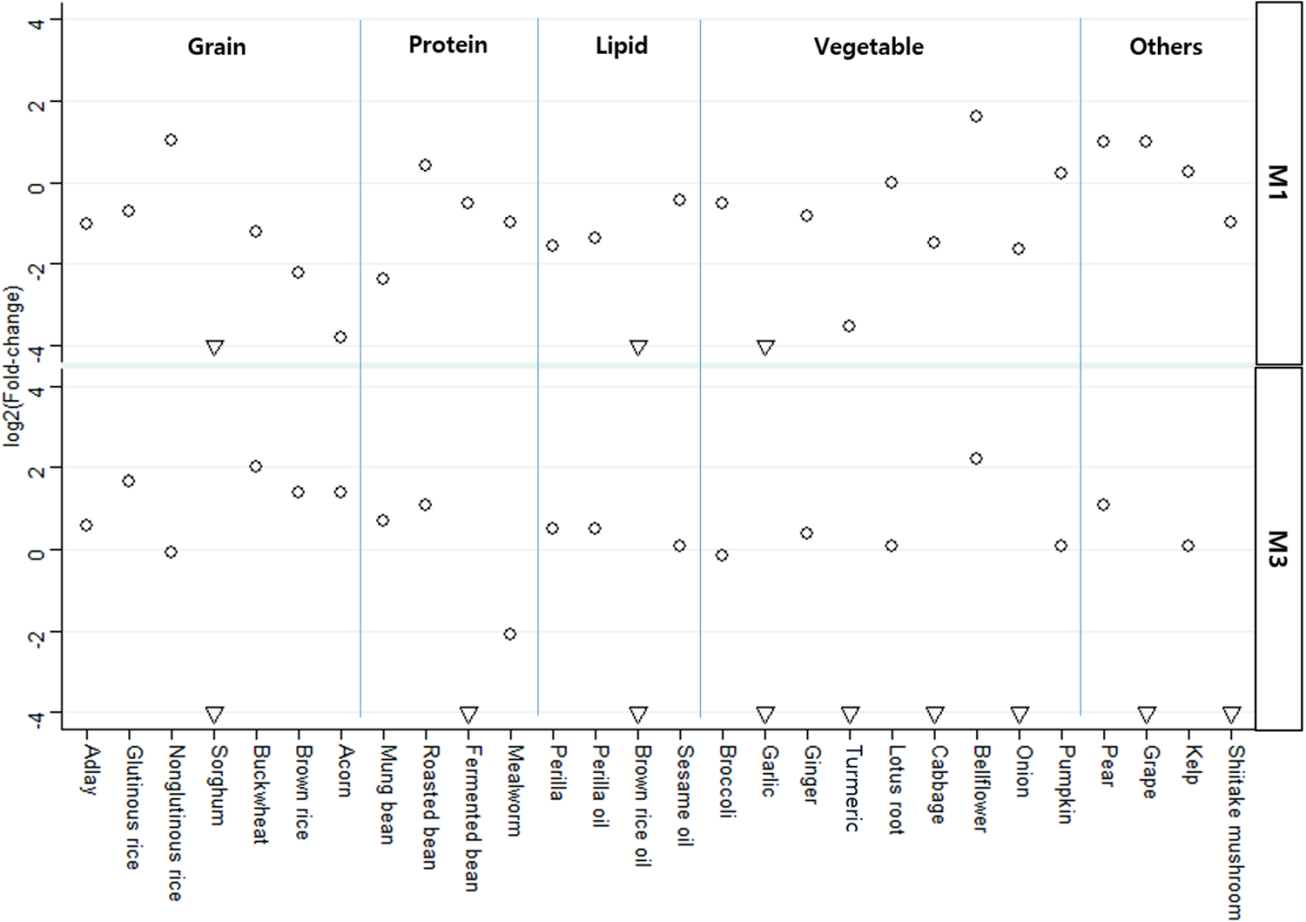
Fold-change in brain tumor (BT) risk between high fat diet (HFD) and HFD+ fed mouse groups. The M1 (stepwise selection) and M3 (LEfSe) BT diagnostic models developed in this study were applied to mice fed either an HFD or an HFD with an additional food item (HFD+). The fold-change of the fitted value of BT risk between the HFD and HFD+ groups was plotted for each dietary food as well as each diagnostic model.

To analyze the relationship between EV microbiome-associated BT risk and diet, genus-level relative abundances of the serum EV microbiome of mice fed an HFD and mice fed an HFD supplemented with an additional food item (HFD+) were fitted to the M1 and M3 models. Serum bacterial EV composition differed significantly between the different dietary groups, and the fitted value obtained through each model was also subsequently altered. However, the difference between the dietary effect of the regular chow diet and the HFD on brain tumor risk was not significant (Supplementary Fig. 5). The absolute value and negative/positive values of fold-changes in the fitted model values between the HFD and HFD+ groups differed between the M1 and M3 models. BT risk was drastically decreased through the addition of sorghum, brown rice oil, and garlic to the HFD with under 0.0625-fold changes in the fitted model value obtained through both M1 and M3 models. In addition, supplementation of fermented bean, mealworm, turmeric, cabbage, onion, and shiitake mushroom reduced BT risk in HFD-fed mice. Conversely, BT risk was increased by the addition of bellflower and pear. The cooking process of the dietary supplements also affected the alteration of EV microbiome-associated BT risk. The addition of roasted beans increased BT risk, while fermented beans drastically decreased BT risk. In addition, lipids derived from brown rice were shown to significantly reduce BT risk in both diagnostic models (Fig. 7).

## Discussion

In this study, we established significant differences between the serum EV microbiome of healthy subjects and BT patients. Based on this analysis, we developed diagnostic models using serum microbial EV biomarkers with high model strength and accuracy. Recently, studies on the relationship between the gut microbiome and brain have increased. Previous studies have shown that the gut microbiome is associated with brain health, especially in a variety of neurological disorders^17,19^. While the relationship between BT growth and development and neurological disorders is uncertain, BTs can place a significant burden on patient vision, mobility, speech, and other cognitive functions^20^. Thus, the gut microbiome might be associated with overall brain function, particularly through the gut–brain axis. The ways in which the gut microbiome affects brain health include excessive stimulation of the immune system by bacterial components such as LPS, antigenic proteins, neurotoxic metabolites released by bacterial enzymes, hormones and neurotransmitters released by gut microbes, and direct stimulation of gut bacteria through the vagus nerve^19^. We suggest that bacterial EVs may be a key mechanism of interaction between the gut microbiome and the brain because nanosized microbial EVs can transverse the BBB and deliver a variety of molecules, including LPS, proteins, nucleic acids, and metabolites, to host cells^21–23^. While commensal gut bacteria are generally restricted to the gastrointestinal system, their EVs can transverse the gut epithelial lining and enter systemic circulation in the bloodstream^10^. BBB-penetrating microbial EVs warrant increased investigation, as their cargo, such as sRNAs, can promote neuroinflammation via TNF-alpha activation^24^. In light of these findings, we suggest that the gut microbiome can affect brain health via circulating EVs and that bacterial EVs in blood could be powerful biomarkers in the assessment of brain disease.

In this study, we analyzed bacterial EVs in blood and tissue as BT biomarkers for the first time. We found that the distribution of bacterial EVs in both blood and tissue samples was significantly different between HC and BT patients. At the genus level, the number of significantly different bacterial EVs between HCs and BT patients was higher in blood than in tissue samples. Through this analysis, we revealed that the *Dialister* and *E. rectale* of BT patients are significantly decreased in both blood and tissue in comparison to the respective levels in HC subjects. However, *Lachnospiraceae NK4A136* was significantly increased in the blood of BT patients, whereas it was decreased in BT-derived tissue. A previous study showed that the abundance of *Dialister*, *E. rectale*, and *Lachnospiraceae NK4A136* in the gut is related to several neurological disorders. For example, *E. rectale* is lower in patients with amyloid-positive and amyloid-negative mild cognitive impairment (MCI)^17^, while *Dialister* has been shown to be diminished in individuals with autism spectrum disorder, Alzheimer’s disease, and depressive disorder patients compared to the respective levels in healthy subjects^25–27^. In addition, an in vivo study demonstrated that *Lachnospiraceae NK4A136* is reduced in Alzheimer’s disease patients in comparison to the levels in healthy controls^28^. Bacterial EVs are secreted from bacteria as shedding vesicles, ectosomes, and apoptotic bodies^9^. Decreased bacterial populations could lead to a reduction in proliferation activity, leading to fewer secreted EVs; therefore, communication components might also be decreased. However, as bacterial populations are reduced, apoptosis induces the secretion of apoptotic bodies that may cross the intestinal epithelial cell layer and circulate throughout the blood. Therefore, we suggest that the interplay of gut microbiota abundance and the blood EV microbiome has a very complex and potentially nonlinear relationship. Further studies on the role of circulating bacterial EVs in communication between the gut microbiota and various organs should be performed. Additional investigations should focus on verifying the efficacy of bacterial EVs in the treatment and diagnosis of a variety of neurological diseases.

In a previous study, we demonstrated that bacterial EVs in serum had strong potential as diagnostic biomarkers for hepatocellular carcinoma^11^. Following those findings, in this study, we analyzed bacterial EVs in blood as biomarkers for BT diagnosis for the first time. Furthermore, we applied machine learning to analyze blood EV microbiome data for highly accurate BT diagnosis. The results of this study are significant as a novel and accurate diagnostic method for BTs and yielded an AUC over 0.93 in all diagnostic models. Other studies exploring diagnostic applications for BTs used a computer-aided detection method based on magnetic resonance imaging (MRI) with an accuracy of 99%^29^ in addition to a core of iron oxide as a nanoparticle based on MRI contrast agents^15^. Our results showed that bacterial EVs in blood also have strong potential as biomarkers aiding in the detection of BTs that may not be readily detected through conventional imaging methods.

Aside from determining the diagnostic potential of serum EVs in BTs, we also sought to further determine dietary interventions that could reduce BT risk associated with the serum EV microbiome. Dietary habits have significant impacts on gut microbiota activity and community structure that are known to influence health^30^. Here, we found that different diets impacted the composition serum EV microbiome components associated with BTs in mice. Of the dietary supplements tested in this study, sorghum, brown rice oil, garlic, cabbage, and onion were the primary foods that decreased serum EV microbiome-associated BT risk, whereas bellflower increased the risk. Only a few published epidemiological studies have reported the impact of vegetable consumption on glioma risk^31,32^. Specifically, different colored vegetables showed different abilities to decrease glioma risk^33^. Leafy green vegetables and yellow-orange vegetables were significantly related to decreased BT risk, especially glioma, whereas eggs, grains, noncured meat, and citrus fruits were significantly related to increased risk^34^. These previous studies targeted combined diets, such as vegetables, grains, and fruits, rather than specific foods, as these studies were primarily epidemiological studies assessing diet on a large scale. However, this study showed the relation between BT risk and individual foods based on differential serum EV microbiome composition in an in vivo model. Furthermore, the BT risk associated with a certain food can vary according to the applied cooking method. The results of this study suggest that roasting might increase risk and extracted oil (lipid) might decrease risk of BT. However, further study is necessary using other foods and comparisons of precise cooking methods such as cooking time, concentration of ingredients, and cooking environment.

Recently, studies on medicinal food and dietary treatments for neurological diseases have been increasing. For example, supplementation of arginine-rich food with conventional therapeutics has been shown to improve several aspects of stable angina patients’ quality of life and vascular function^35^. Furthermore, consumption of medicinal food containing phosphatide precursors and various cofactors for 12 weeks was demonstrated to improve memory in patients with mild cases of Alzheimer’s disease^36^. Several taxa of bacterial EVs derived from serum that were shown to be significantly decreased in BT patients in this study, *Dialister, Bifidobacterium*, and *E. rectale*, were demonstrated to be significantly increased by certain dietary treatments, including brown rice and whole grain barley, in a previous study^37^. Strategic dietary management could improve the symptoms of brain diseases and enhance treatment efficacy. Dietary management affects human health through various routes, including chemical, nutrient, immunology, and microbiome modulation. Therefore, dietary treatment has vast potential to supplement and enhance neurological disease therapy and medicinal approaches. The dietary intervention findings reported in this study provide a strong foundation for further investigation of dietary treatment to reduce BT risk.

This study has several limitations, including a relatively small sample size and limited clinical information that included only age and sex. In addition, there was a significant difference in age between the BT and HC groups, which could potentially act as a confounding factor in this study. Age may affect the microbiome composition of serum^38^; however, the relation between age and serum microbiome composition has yet to be clearly demonstrated. Further study should determine if the factor of age is a potentially complicating effect and minimize such factors accordingly. In addition, the samples used in this study were drawn solely from a hospital population, which could limit the representativeness and generalization of these results. The diagnostic models developed in this study should be further validated using a larger sample size from a more diverse patient population, and more clinical information should be collected. Another limitation of this study that could act as a confounding factor in our diagnostic models was the lack of control for medication usage, sampling time, diet, and physical activity^39,40^. In the future, we should incorporate drug-naive or drug-free patients, dietary information, and physical activity of patients for greater insight. In addition, more precise exploration of the influence of diet on BT risk is necessary. To thoroughly determine the effect of diet on BT patients, clinical testing should be conducted as well as larger animal brain tumor models. In addition, the cooking method should be more precisely characterized. Alterations in cooking methods such as roasting, fermentation, oil extraction, temperature, time, humidity, and concentration could have different effects. Therefore, future studies should more tightly control these variables and compare various cooking methods of the same food to determine the optimal dietary intervention for BTs. Finally, the exact mechanism through which serum bacterial EVs influence BT development should be verified in the future.

In conclusion, this study provides further evidence to support our expanding understanding of the complex, underexplored role of circulating EVs as biomarkers of chronic disease. By incorporating machine learning and serum EV metagenomics, we developed high-strength BT diagnostic models in a clinical cohort that were then used to determine the efficacy of individual foods to reduce serum microbial EV-BT risk in vivo. Future studies including larger patient cohorts, more detailed clinical information, and efforts to determine the mechanism through which microbial EVs impact brain health should be conducted to validate the results of this study.

## Supplementary information

Supplementary Figures
