# Supplementary material for: Brain tumor diagnostic model and dietary effect based on extracellular vesicle microbiome data in serum
Source: Exp Mol Med. 2020 Sep 16;52(9):1602–13. doi: 10.1038/s12276-020-00501-x (PMC8080813; doi:10.1038/s12276-020-00501-x)

Supplementary Fig. 1. Beta diversity based on PCoA at the a) phylum, b) class, c) order, and d) family levels

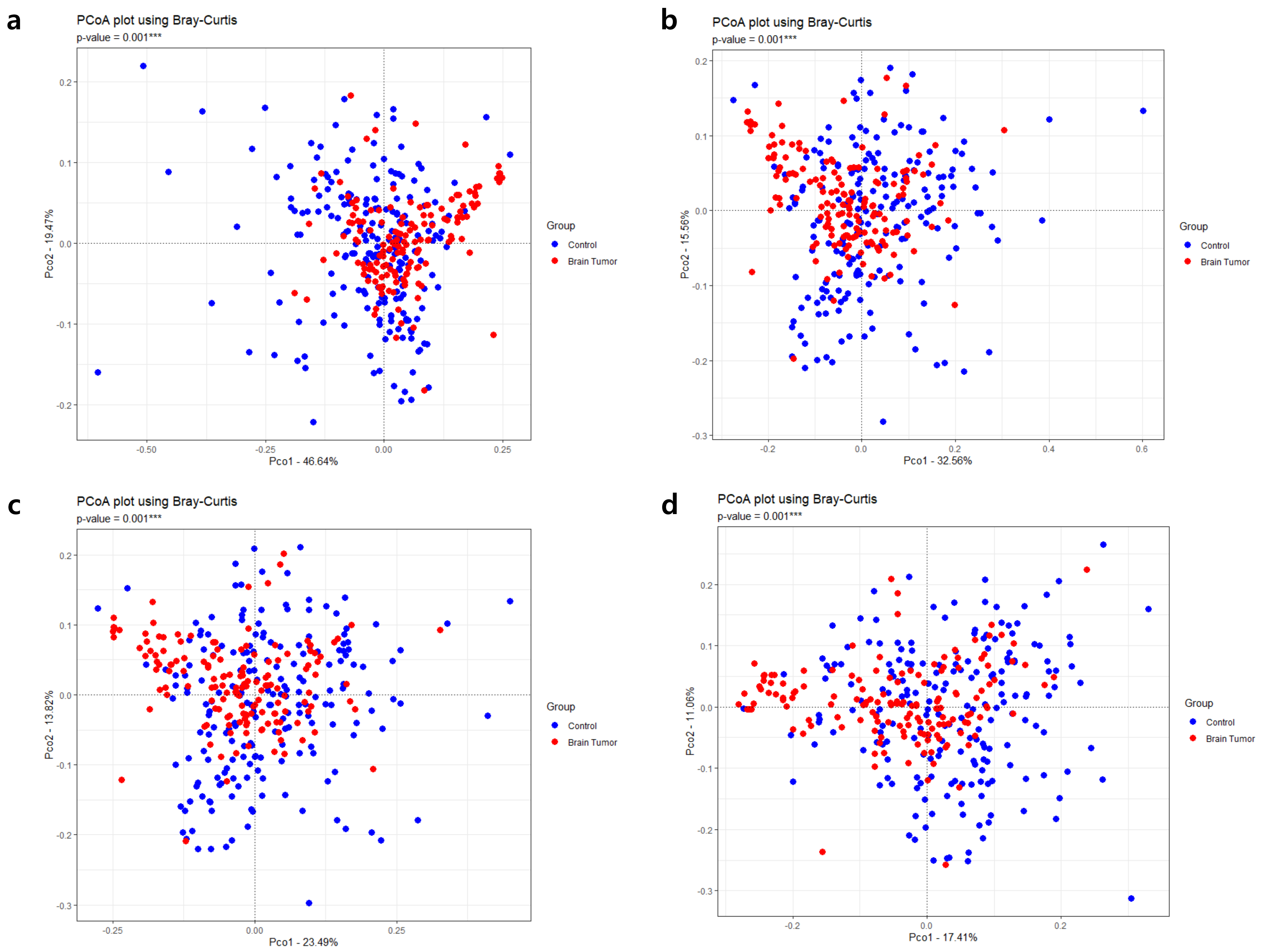

Supplementary Fig. 2. Abundance of microbiome in serum at the a) class, b) order, and c) family levels (**: $p<0.05$, ***: $p<0.01$)

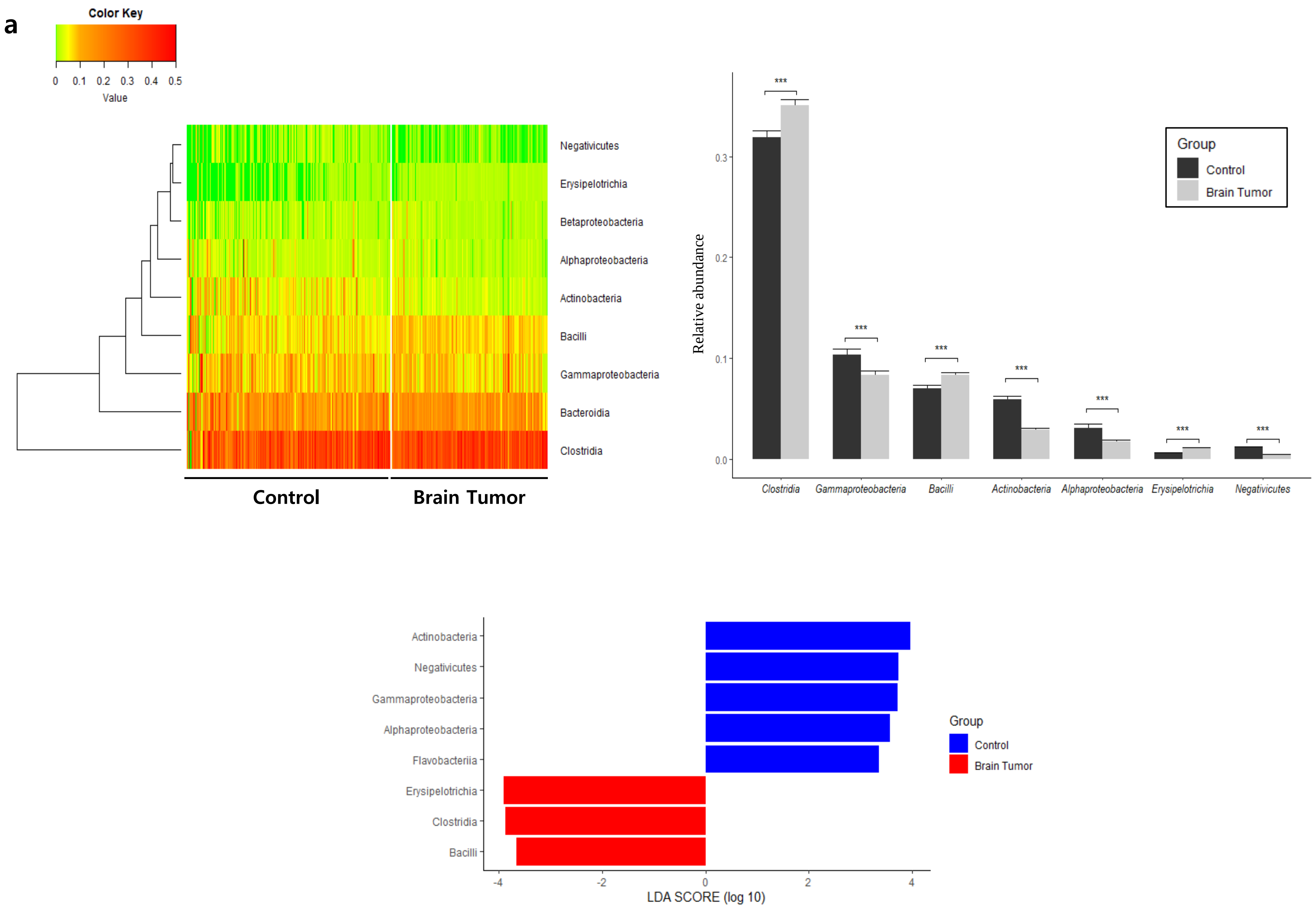

Supplementary Fig. 2. Abundance of microbiome in serum at the a) class, b) order, and c) family levels (**: p<0.05, ***: p<0.01)

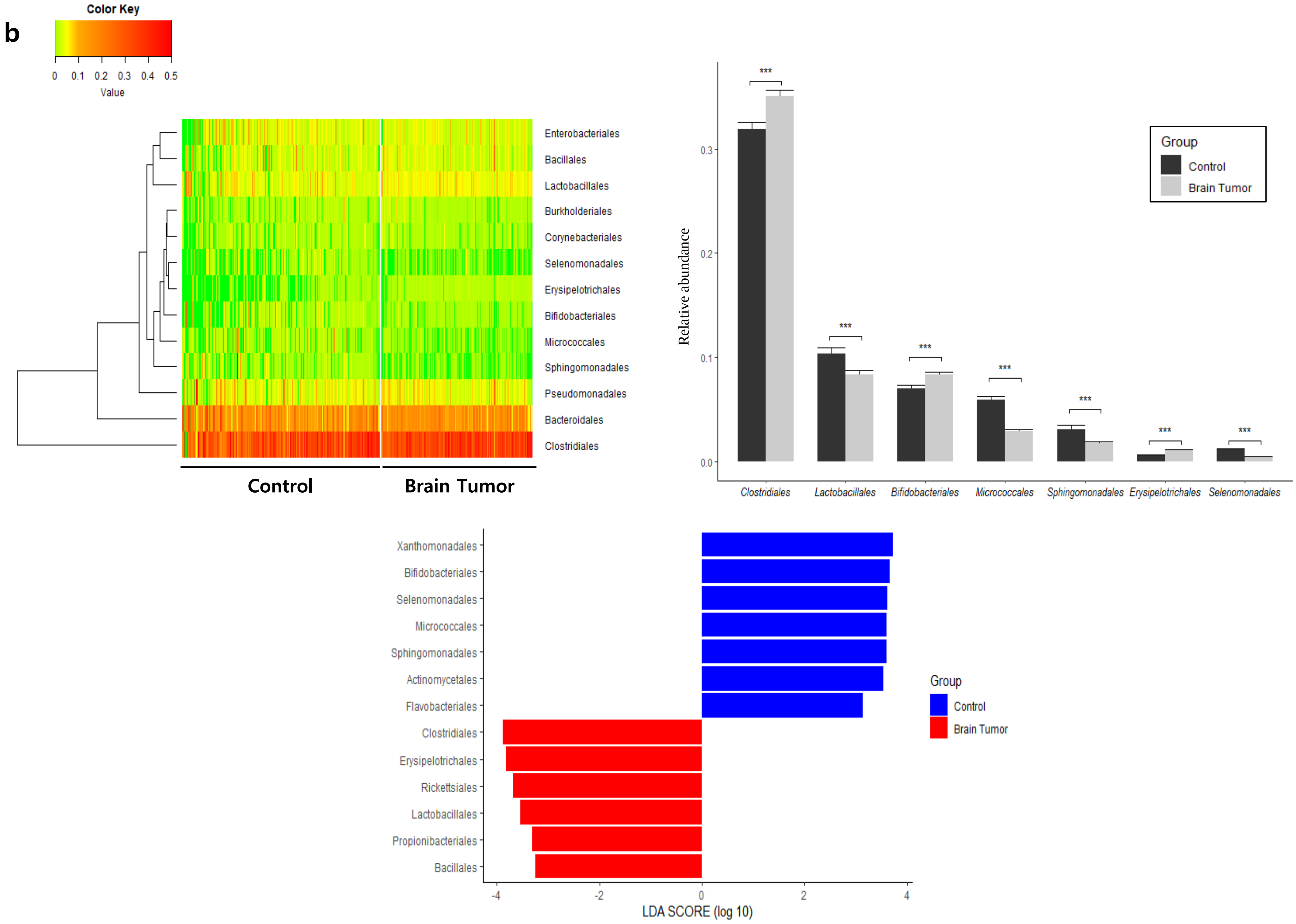

Supplementary Fig. 2. Abundance of microbiome in serum at the a) class, b) order, and c) family levels (**: p<0.05, ***: p<0.01)

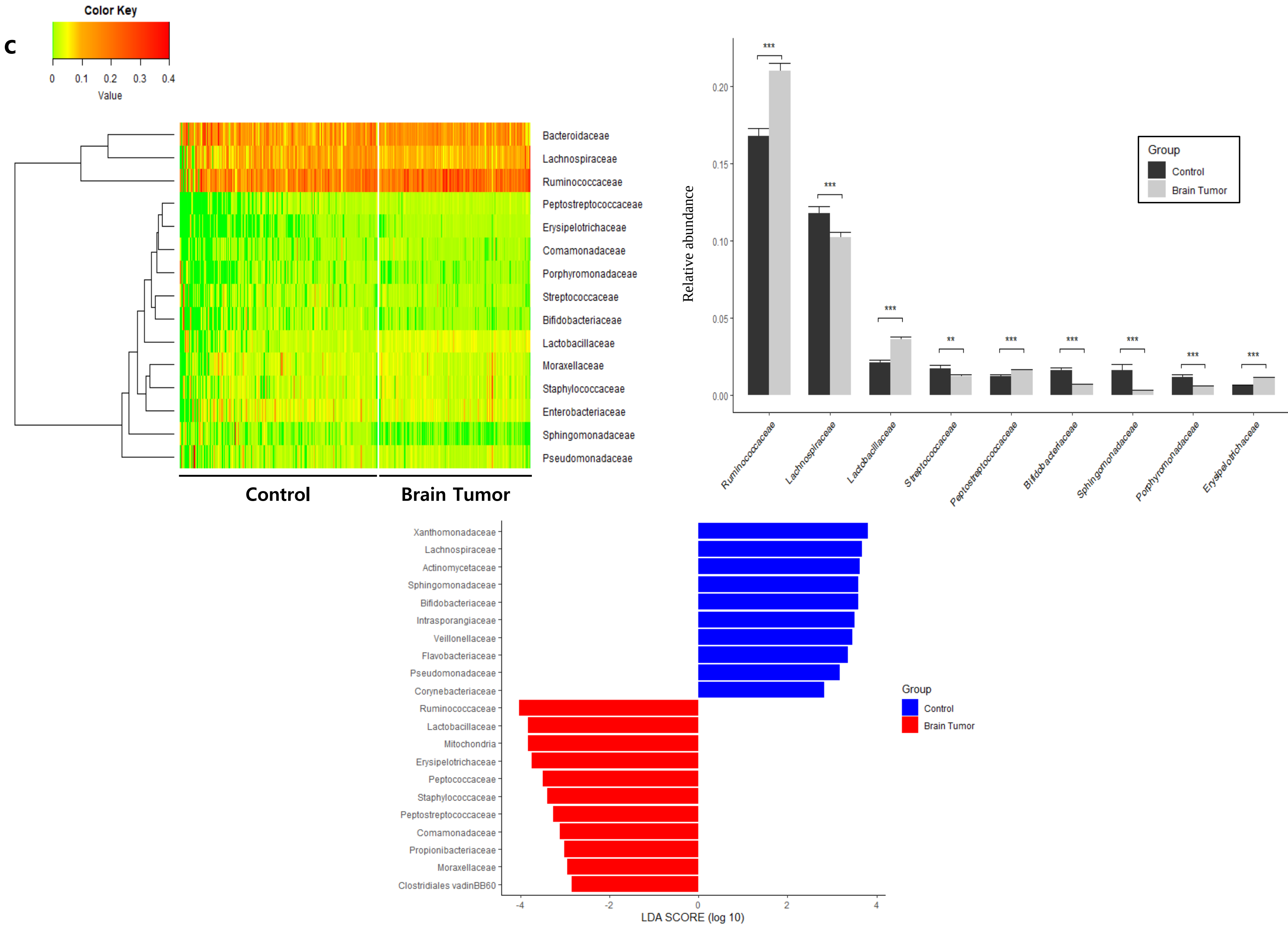

Supplementary Fig. 3. Abundance of microbiome in brain tissue at the a) class, b) order, and c) family levels (**: p<0.05, ***: p<0.01)

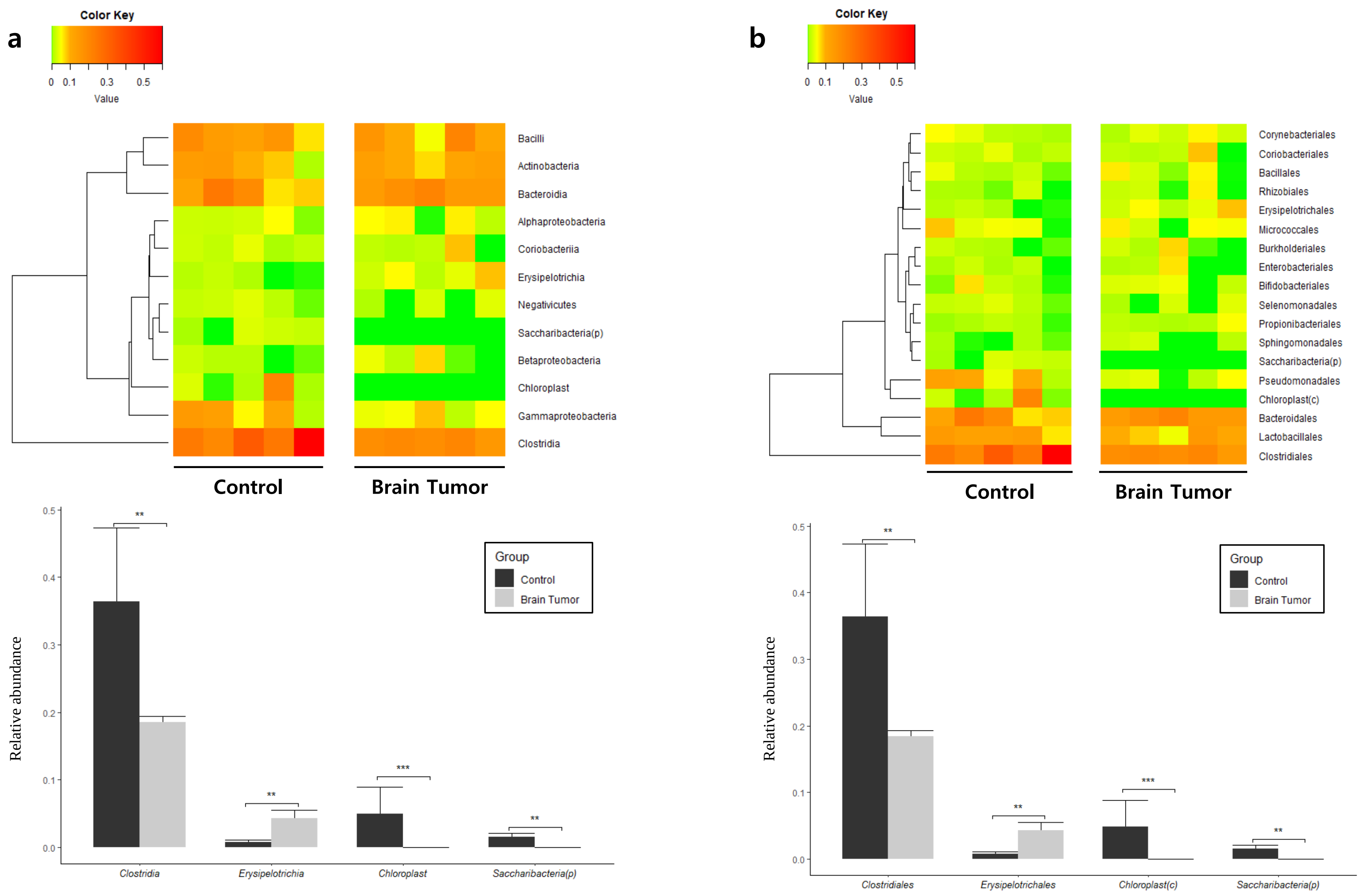

Supplementary Fig. 3. Abundance of microbiome in brain tissue at the a) class, b) order, and c) family levels (**: p<0.05, ***: p<0.01)

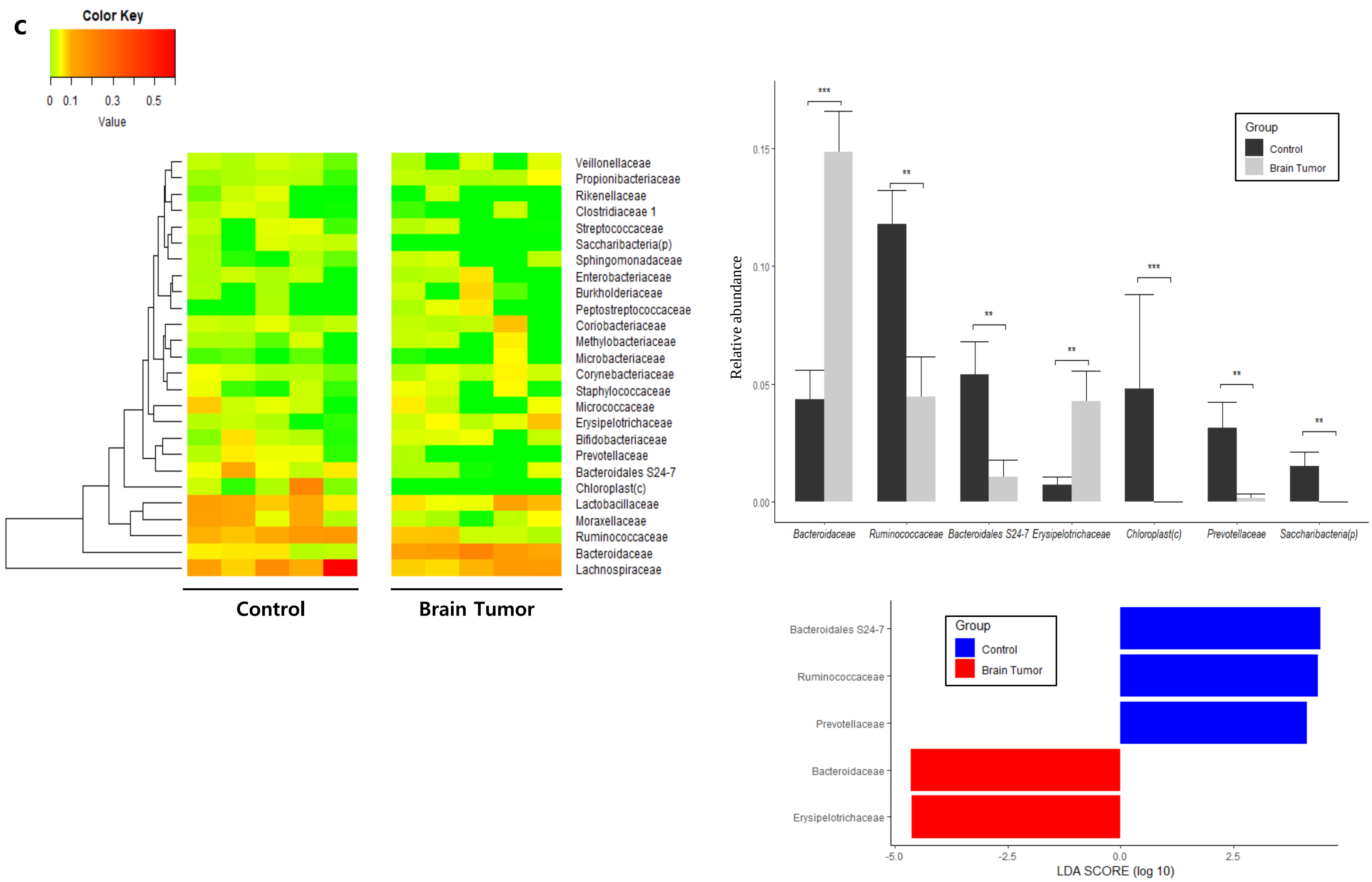

Supplementary Fig. 4. Fold-change between healthy control subjects (HC) and brain tumor patients (BT) in serum and tissue at the a) phylum, b) class, c) order, and d) family level: Red means significant in serum, Green means significant in tissue, and Blue means significant in both serum and tissue

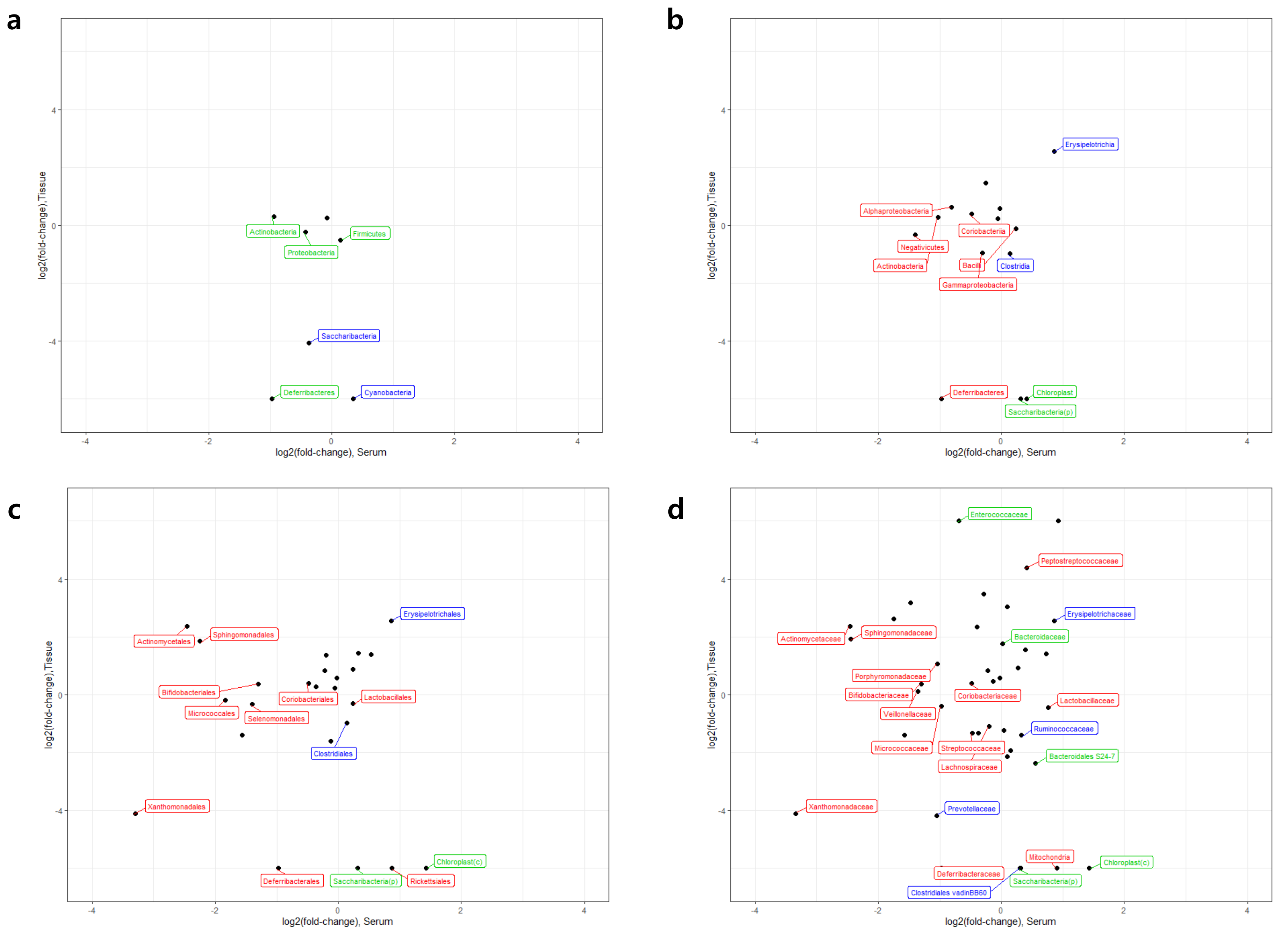

Supplementary Figure 5. in vivo test for brain tumor risk between regular chow-fed and high fat diet fed mouse groups

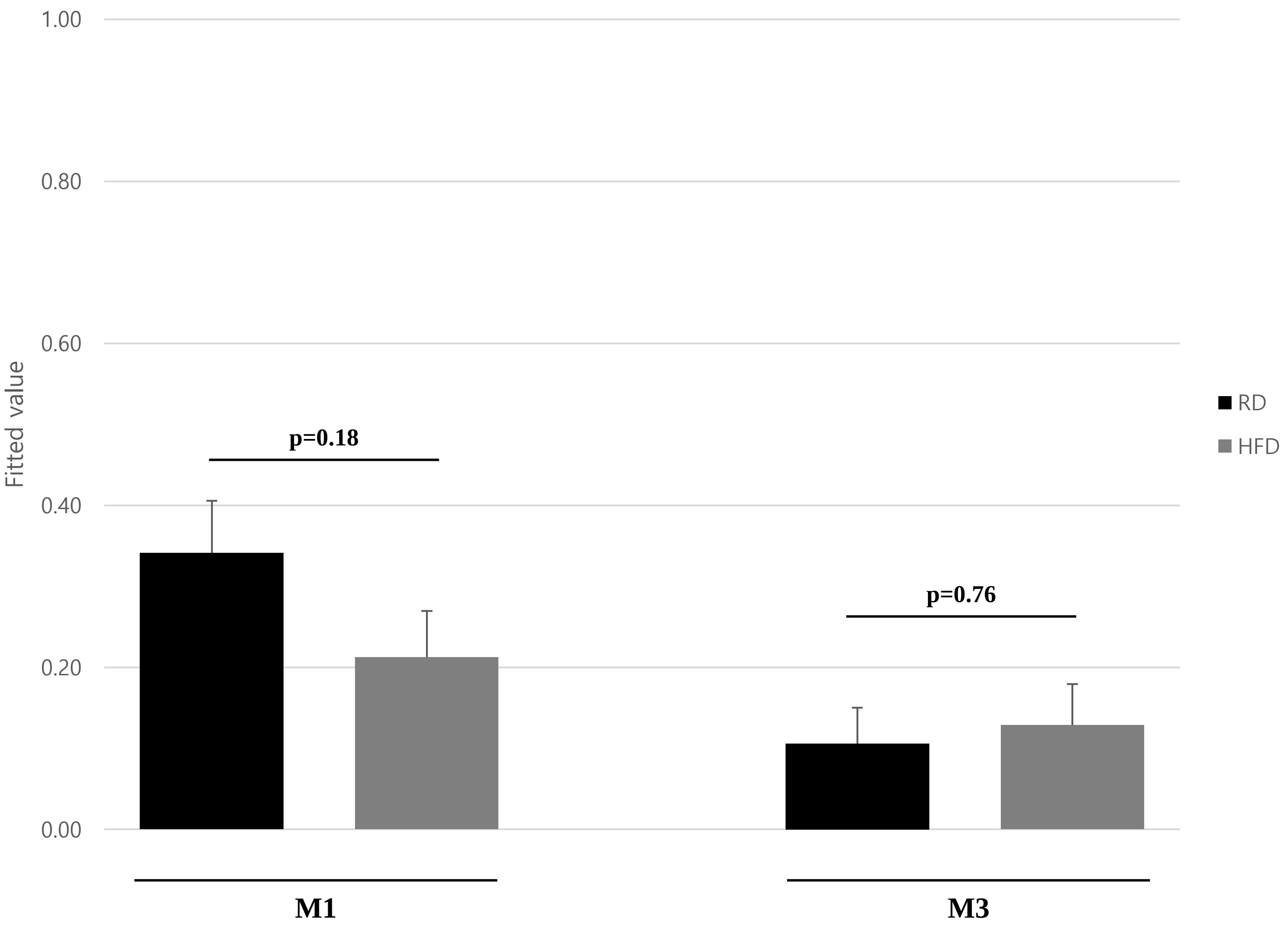

Supplement: Supplementary file 1 — Supplementary Figures [file 12276_2020_501_MOESM1_ESM.pdf]
